# Phase 1 trial for treatment of COVID‐19 patients with pulmonary fibrosis using hESC‐IMRCs

**DOI:** 10.1111/cpr.12944

**Published:** 2020-10-26

**Authors:** Jun Wu, Xing Zhou, Yuanqing Tan, Liu Wang, Tianda Li, Zhongwen Li, Tingting Gao, Jiaqi Fan, Baojie Guo, Wei Li, Jie Hao, Xianguang Wang, Baoyang Hu

**Affiliations:** ^1^ Department of National Stem Cell Resource Center Institute of Zoology Chinese Academy of Sciences Beijing China; ^2^ Institute for Stem Cell and Regeneration Chinese Academy of Sciences Beijing China; ^3^ State Key Laboratory of Stem Cell and Reproductive Biology Institute of Zoology Chinese Academy of Sciences Beijing China; ^4^ Wuhan Jinyintan Hospital Wuhan China; ^5^ University of Chinese Academy of Sciences Beijing China

**Keywords:** COVID‐19, CT, IMRCs, pulmonary fibrosis

To the Editor:

Pulmonary fibrosis is a severe complication in COVID‐19 patients. Based on our previous success with stem cell therapy of acute lung injury in mouse models and 2 COVID‐19 patients in a pilot compassionate use study,^1^ we describe a Phase 1 clinical trial where we treated COVID‐19 patients with pulmonary fibrosis using human embryonic stem cell–derived immunity‐ and matrix‐regulatory cells (hESC‐IMRCs) during the SARS‐CoV‐2 outbreak in Wuhan City.

Based on our recruitment criteria, we identified 27 COVID‐19 patients who demonstrated pulmonary fibrosis pathology (Figure S1). All subjects had remained SARS‐CoV‐2–positive for more than three weeks. The median age of the patients was 66.5 years, of which 70% were men (Table 1). Patients with confirmed COVID‐19 were diagnosed with pulmonary fibrosis using chest CT scans. A total of 20 patients were inpatients, while the other 7 subjects were discharged patients who still presented respiratory symptoms due to pulmonary fibrosis. Of the 20 inpatients, 10 were classified as severe/critical cases while another 10 were classified as moderate cases. Of the 20 inpatients who had pulmonary fibrosis and experienced shortness of breath at rest or exercise, 16 of 20 were supported by continuous oxygen therapy, of whom 1 depended on high‐flow oxygen therapy. Another 4 of 20 inpatients also had pulmonary fibrosis and various respiratory symptoms, but only needed oxygen therapy after exercise. The 7 discharged patients presented respiratory symptoms only when they walked quickly.

**Table 1:** Characteristics of 27 COVID‐19 patients with pulmonary fibrosis

Characteristics	Total (N = 27)	Severe/Critical inpatients (N = 10)	Moderate inpatients (N = 10)	Discharged patients (N = 7)
Median age—year (IQR)	66.5(38‐79)	67.8 (49‐79)	61.78(46‐77)	51.5 (38‐62)
Male sex—no. (%)	19 (70)	6 (60)	4 (40)	7 (100)
Risk factor—no. (%)				
Hypertension	9 (33.3)	6 (60)	3 (30)	0
Diabetes mellitus	7 (25.9)	5 (50)	2 (20)	0
Signs and symptom onset				
Chest pain	1 (27)	1 (10)	0	0
Fever	22 (27)	9 (10)	7 (10)	6 (7)
Cough, shortness of breath or respiratory distress	20 (27)	7 (10)	7 (10)	6 (7)
Myalgia	1 (27)	0	1 (10)	0
Diarrhoea	2 (27)	1 (10)	1 (10)	0
Signs and symptoms at the first round of IMRC infusion				
Fever	0	0	0	0
Cough, shortness of breath or respiratory distress	27 (27)	10（10）	10 (10)	7 (7)
Myalgia	1 (27)	0	1 (10)	0
High flow of oxygen	1 (20)	1 (10)	0	—
Low flow of oxygen	15 (10)	9 (10)	6 (10)	—
Inpatient days (IQR)	59 (43‐74)	67 (48‐74)	59 (43‐63)	—
Days of disease process (IQR)	75 (43‐98)	74.8 (43‐77)	65 (51‐71)	84.5 (60‐98)
Inpatient days after IMRC infusion (IQR)	10.3 (5‐28)	10.8 (7‐15)	9 (5‐28)	—
Chest CT findings—no. (%)				
Opacities in both lungs	19 (70.3)	9 (90)	6 (60)	4 (57.1)
Pulmonary consolidation	3 (11.1)	1 (10)	0	2 (18.6)
Focal opacities	21 (77.8)	7 (70)	6 (60)	6 (85.7)
Gridded	16 (59.3)	7 (70)	6 (60)	3 (42.9)
Chest CT findings after cell infusion	All the subjects’ lung fibrotic lesion areas had diminished as observed from their chest CT scans after hESC‐IMRC treatment at different times of follow‐up.			
Previous treatments—no. (%)				
Fibrinolytic agent	0	0	0	0
Glucocorticoids	3 (11.1)	1 (10)	2 (10)	0
Immunomodulatory drugs	5 (18.5)	2 (10)	3 (10)	0
Other trials—no.				
Convalescent plasma	3 (27)	3 (10)	0	0
Traditional Chinese Medicine	2 (27)	1 (10)	1 (10)	0
Remdesivir	3 (27)	2 (10)	1 (10)	0
Bismuth potassium citrate	1 (27)	1 (10)	0	0
Rounds of IMRC infusion—no.				
Once	1	—	1	—
Twice	25	10	8	7
Thrice	1	—	1	—

Abbreviation: IQR, interquartile range.

After obtaining informed consent and approval, all 27 patients received intravenous transfusion of hESC‐IMRCs at a dose of 3 × 10^6^ cells/kg of body weight. 25 of the 27 patients received intravenous transfusion of hESC‐IMRCs twice, whereas the other 2 patients received intravenous infusion of hESC‐IMRCs once and thrice, respectively. The patient who received three hESC‐IMRC infusions was classified as a moderate case. His clinical symptoms had disappeared 10 days after the first hESC‐IMRC infusion, but remained SARS‐CoV‐2–positive until 28 days after treatment, upon which he was discharged. We proceeded to follow up with all the patients 84 days after the first hESC‐IMRC treatment by either re‐examination within the hospital or interviews over the phone.

The 7 discharged patients could exercise with moderate intensity without significant symptoms upon 56 days after the hESC‐IMRC treatment. Their lung fibrotic lesion areas had significantly decreased as observed from their chest CT scans (Figure S1C). We collected chest CT images from 3 of the 20 inpatients 7 days after treatment (Figure S1A‐B, Patients #5, #20 and #22). The chest CT images also showed that their fibrosis lesions had improved. Chest CT scans were performed for 2 of the 20 inpatients 28 days after hESC‐IMRC infusion, and their lung fibrotic lesion areas were also found to have decreased significantly (Figure S1A, Patient #10, #23). One of the 20 inpatients was dropped due to withdrawal of consent from the family. For the remaining 14 of 20 inpatients, their lung fibrotic lesion areas were also significantly diminished by 84 days after treatment (Figure S1).

None of the treated patients suffered any adverse events or abnormal responses related to cell therapy. All haematological and clinical chemistry parameters remained in the normal range (Table S1). In addition, no tumour markers were detected in the serum, demonstrating that hESC‐IMRCs were safe for intravenous infusion.

In this series of COVID‐19 patients with pulmonary fibrosis, there were demonstrable improvements in many clinical symptoms within a short period after hESC‐IMRC transfusion (Table 1). Our cell therapy for lung fibrosis is safe for subjects in the medium term, for up to 84 days. Long‐term safety will be observed in long‐term follow‐up at a later stage. Due to the special emergency presented by the COVID‐19 outbreak in Wuhan, it should be noted that 7 of the 10 severe/critical cases had already undergone other clinical trials and 28 days of follow‐up prior to this Phase 1 trial, but had still remained positive for pulmonary fibrosis. However, 27 of 27 patients displayed clinical improvements within 84 days after treatment with our hESC‐IMRC therapy. In this Phase 1 trial, we have demonstrated that hESC‐IMRCs are safe for intravenous infusion in the medium term, and our preliminary results showed efficacy for pulmonary fibrosis in COVID‐19 patients. In order to further evaluate the clinical efficacy of this approach, we are now performing a multicentre randomized placebo‐controlled Phase 2/3 trial.

## CONFLICT OF INTEREST

The authors declare that there is no conflict of interest that could be perceived as prejudicing the impartiality of the research reported.

## AUTHOR CONTRIBUTIONS

BH, XW and JH conceived the project. ZL, TG and BG provided the testing results of IMRCs. TL, FJ, XZ and WL analysed clinical case. JW, XZ, YT and LW designed the research and wrote the manuscript with help from all of the authors. JW, XZ, YT and LW contributed equally to this letter. BH, XW and J.H are the corresponding authors of this letter. All authors read and approved the final manuscript.

## ETHICAL APPROVAL

The study is approved by the Ethics Committee of Jinyintan Hospital, Wuhan, China.

## CLINICAL TRIAL REGISTRATION

This clinical trial was registered with the National Medical Products Administration of China.

## Supporting information

Figure S1A

Figure S1B

Figure S1C

Table S1

## Data Availability

The data that support the findings of this study are available from the corresponding author upon reasonable request.
