# Supplementary figures and images for: Phase 1 trial for treatment of COVID‐19 patients with pulmonary fibrosis using hESC‐IMRCs
Source: Cell Prolif. 2020 Oct 26;53(12):e12944. doi: 10.1111/cpr.12944 (PMC7645925; doi:10.1111/cpr.12944)

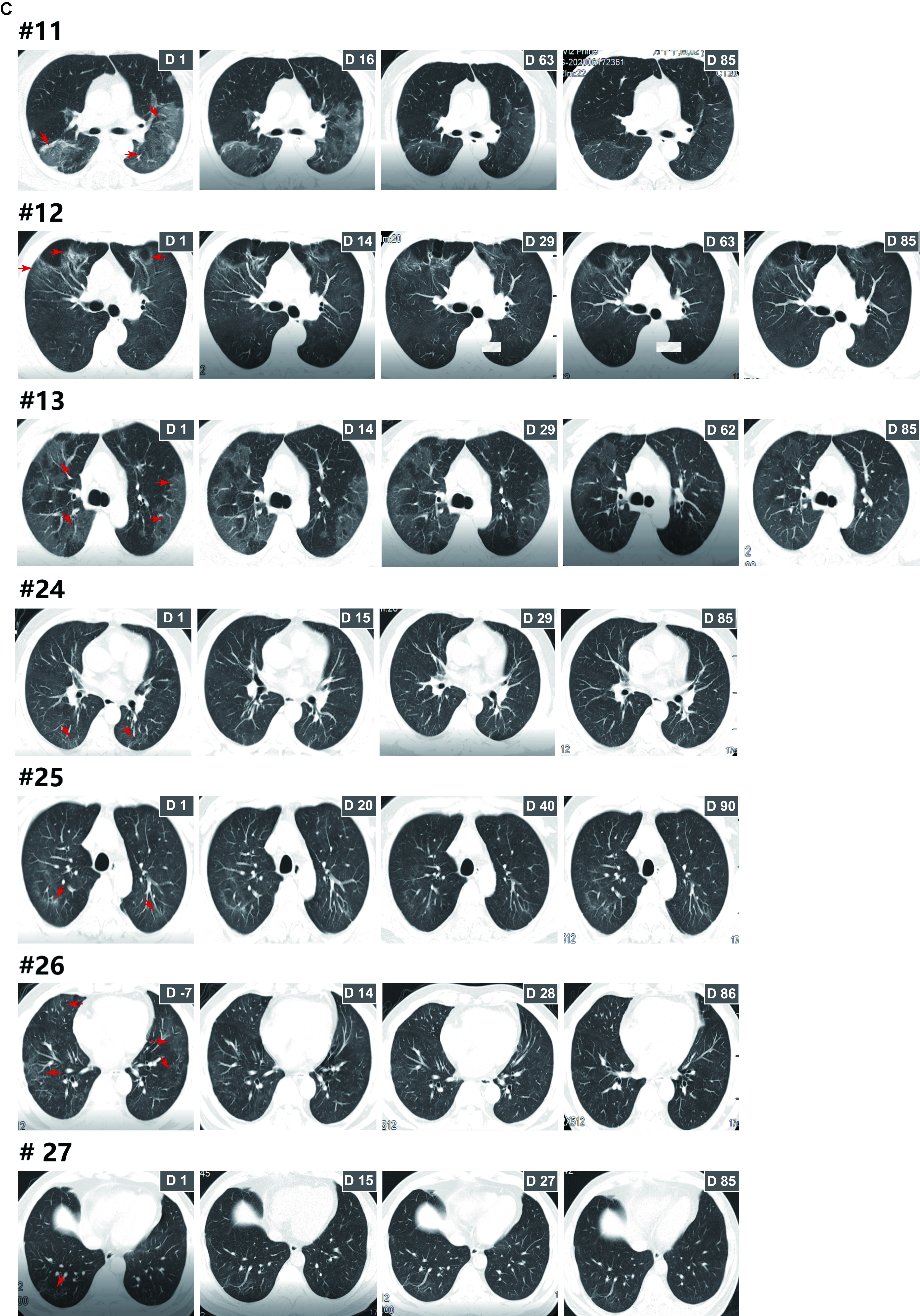

Supplement: Supplementary file 3 — Figure S1C [file CPR-53-e12944-s003.tif]
