# Supplementary material for: Phase 1 trial for treatment of COVID‐19 patients with pulmonary fibrosis using hESC‐IMRCs
Source: Cell Prolif. 2020 Oct 26;53(12):e12944. doi: 10.1111/cpr.12944 (PMC7645925; doi:10.1111/cpr.12944)
Supplement: Supplementary file 4 — Table S1 [file CPR-53-e12944-s004.doc]

**Supplementary Appendix**

This appendix has been provided by the authors to give readers additional information on their work.

**Supplementary** **Figure 1.** Chest CT scans

**Supplementary Table 1** Clinical Laboratory Results

**FIGURE LEGENDS**

**Supplementary Figure 1 Chest CT scans of COVID-19 patients with pulmonary fibrosis, before and after hESC-IMRC treatment.** [A] Chest CT scans for the severe cases. [B] Chest CT scans for critical patients. [C] Chest CT scans for the discharged patients.

The red arrowheads indicate pulmonary lesions at baseline

| **Supplementary Table 1 Clinical Chemistry Laboratory Results** | | | | | | | | | | |
| --- | --- | --- | --- | --- | --- | --- | --- | --- | --- | --- |
|  |  | **CRP (mg/L)** | **White blood cell count (10^9^/L)** | **Neutrophils (%)** | **Lymphocytes (%)** | **ALT (U/L)** | **AST (U/L)** | **DBIL (umol/L)** | **TBIL (umol/L)** | **Creatinine (umol/L)** |
| **Patient** | **Time** | **＜3** | **3.5-9.5** | **40-75** | **20-50** | **9-50** | **15-40** | **＜7** | **5-21** | **57-97** |
| **#1** | baseline | 0.4 | 5.78 | 61.3 | 22.8 | 34 | 25 | 5.4 | 16.9 | 45.6 |
|  | D7 | 0.5 | 4.92 | 67.2 | 19 | 23 | 16 | 5.1 | 17.4 | 48 |
| **#2** | baseline | 3 | 13.2 | 65.7 | 22.5 | 14 | 20 | 4.5 | 16.7 | 37.8 |
|  | D7 | 3.2 | 9.42 | 53.7 | 31.5 | 15 | 18 | 3.5 | 13 | 42.2 |
| **#3** | baseline | 3.1 | 8.01 | 79.2 | 14.3 | 19 | 19 | 4.5 | 13.9 | 73.5 |
|  | D7 | 39.3 | 6.3 | 77.7 | 15.7 | 25 | 17 | 3.3 | 13.3 | 75.9 |
| **#4** | baseline | 80.5 | 9.02 | 68.1 | 20.1 | 29 | 18 | 4 | 10.9 | 66.3 |
|  | D7 | 7.8 | 8.73 | 56.7 | 33.1 | 15 | 14 | 2.7 | 10.7 | 70.3 |
| **#5** | baseline | 2.2 | 5.45 | 74.5 | 12.1 | 14 | 25 | 3.1 | 8.9 | 90.6 |
|  | D7 | 10.2 | 5.44 | 75.3 | 11.9 | 10 | 19 | 3.2 | 8.9 | 91.8 |
| **#6** | baseline | 2.3 | 6.56 | 53.5 | 32.9 | 14 | 17 | 2.8 | 8.7 | 72.9 |
|  | D7 | 0.6 | 5.04 | 57.8 | 30.1 | 9 | 11 | 3 | 10.3 | 64.8 |
| **#7** | baseline | 18.8 | 4.49 | 57.3 | 28.5 | 24 | 29 | 3.7 | 13.7 | 55.4 |
|  | D7 | 0.5 | 4.69 | 52.2 | 37.7 | 17 | 20 | 2.4 | 9 | 51.2 |
| **#8** | baseline | 0.8 | 5.49 | 54.2 | 34.1 | 31 | 26 | 3.6 | 9.9 | 39.5 |
|  | D7 | 2.3 | 7.8 | 49.7 | 41.2 | 25 | 28 | 3.4 | 11 | 40 |
| **#9** | baseline | 0.5 | 4.65 | 57.6 | 30.7 | 32 | 24 | 4.6 | 13.4 | 45.7 |
|  | D7 | 0.8 | 6.18 | 70.4 | 21.3 | 39 | 23 | 4.4 | 13.1 | 40.8 |
| **#10** | baseline | 1.6 | 4.33 | 53.8 | 32.8 | 39 | 26 | 4.1 | 10 | 42.3 |
|  | D7 | 0.7 | 5.78 | 46.7 | 39.5 | 61 | 44 | 3.3 | 12.2 | 53.8 |
| **#11** | baseline | 0.8 | 4.96 | 60.3 | 31.9 | 21 | 23 | 3.1 | 8.7 | 75.4 |
|  | D7 | 0.3 | 7.13 | 65.8 | 26.6 | 15 | 23 | 3.1 | 8.3 | 72.3 |
| **#12** | baseline | 0.6 | 4.17 | 60.4 | 31.7 | 20 | 22 | 3.7 | 10.8 | 62.1 |
|  | D7 | 0.5 | 5.08 | 64.3 | 28.3 | 28 | 23 | 3.6 | 9.8 | 60.2 |
| **#13** | baseline | 2.4 | 5.24 | 61.6 | 31.5 | 54 | 39 | 4.5 | 11.6 | 62.5 |
|  | D7 | 0.6 | 5.41 | 67.1 | 25.6 | 72 | 44 | 4.3 | 10.9 | 65.1 |
| **#14** | baseline | 0.1 | 3.17 | 45.6 | 43.6 | 27 | 25 | 5.1 | 24.8 | 49.7 |
|  | D7 | 2.6 | 4.67 | 52.3 | 35.6 | 16 | 17 | 5.7 | 23 | 42.2 |
| **#15** | baseline | 0.2 | 7.57 | 63.1 | 16 | 32 | 32 | 5.8 | 21.9 | 60 |
|  | D7 | 1.5 | 8.96 | 58.8 | 19.6 | 24 | 18 | 5.1 | 13.8 | 51.2 |
| **#16** | baseline | _ | 5 | 63 | 24.5 | 18 | 20 | 2.1 | 4 | 54 |
|  | D7 | 0.5 | 5.61 | 55.6 | 34 | 16 | 23 | 3.7 | 12.2 | 59 |
| **#17** | baseline | 1.4 | 4.49 | 54.7 | 34.2 | 21 | 27 | 7.4 | 27.8 | 65.1 |
|  | D7 | 0.3 | 3.77 | 54.7 | 34.9 | 19 | 18 | 3.8 | 10.07 | 61.6 |
| **#18** | baseline | 0.7 | 8.93 | 61.2 | 30.3 | 12 | 29 | 2.6 | 9.7 | 39 |
|  | D7 | 0.3 | 6.8 | 48.7 | 43.5 | 9 | 26 | 3.3 | 10.2 | 39 |
| **#19** | baseline | 0.5 | 5.46 | 59 | 29.4 | 20 | 21 | 4.9 | 16.6 | 45.2 |
|  | D7 | 0.3 | 7.96 | 51.1 | 36 | 16 | 18 | 4.6 | 13.2 | 50.2 |
| **#20** | baseline | 1.7 | 5.33 | 38.5 | 48.2 | 18 | 21 | 3.5 | 9.8 | 42.9 |
| **Supplementary Table 1 (Continued)** | | | | | | | | | | |
|  |  | **CRP (mg/L)** | **White cell count (10^9/L)** | **Neutrophils (%)** | **Lymphocytes (%)** | **ALT (U/L)** | **AST (U/L)** | **DBIL (umol/L)** | **TBIL (umol/L)** | **Creatinine (umol/L)** |
| **Patient** | **Time** | **＜3** | **3.5-9.5** | **40-75** | **20-50** | **9-50** | **15-40** | **＜7** | **5-21** | **57-97** |
|  | D7 | 1.4 | 5.19 | 51.1 | 36.6 | 16 | 20 | 3.7 | 7.8 | 40.6 |
| **#21** | baseline | 10.1 | 8.53 | 53.2 | 36.1 | 8 | 17 | 3.9 | 11.3 | 43.9 |
|  | D7 | 6.9 | 10.55 | 52.3 | 38.7 | 11 | 21 | 5 | 12.8 | 44.2 |
| **#22** | baseline | 13.3 | 5.45 | 47.1 | 36.6 | 21 | 29 | 2.9 | 10.9 | 109.6 |
|  | D7 | 3 | 5.51 | 53.2 | 34.1 | 24 | 24 | 3.5 | 9.8 | 118 |
| **#23** | baseline | <0.1 | 9.7 | 70.9 | 18.3 | 7 | 66 | 11.8 | 14.9 | 49.4 |
|  | D7 | <0.1 | 5.14 | 64.6 | 25.8 | 9 | 17 | 6.6 | 20.8 | 54.3 |
| **#24** | baseline | 0.7 | 6.11 | 74 | 18.4 | 38 | 38 | 5 | 12.9 | 927 |
|  | D7 | 0.8 | 5.06 | 70.1 | 20.5 | 36 | 30 | 5 | 14 | 93.6 |
| **#25** | baseline | 0.3 | 6.1 | 57.9 | 33.7 | 25 | 19 | 5 | 13.9 | 58.8 |
|  | D7 | 0.3 | 0 | 0 | 0 | 24 | 21 | 4 | 13 | 62.9 |
| **#26** | baseline | 0.9 | 7.6 | 57.2 | 31.6 | 16 | 24 | 4.5 | 9.4 | 99.5 |
|  | D7 | 1.2 | 5.58 | 63.7 | 21.5 | 16 | 24 | 3.4 | 9.2 | 99.1 |
| **#27** | baseline | 0.6 | 5.82 | 69.8 | 20.6 | 30 | 26 | 4.2 | 13.7 | 62.2 |
|  | D7 | 0.4 | 5.68 | 63 | 26.7 | 18 | 21 | 4 | 13 | 57.6 |

Note: CRP, C Response Protein; ALT, Alanine Aminotransferase; AST, Aspartate aminotransferase; TBIL, Total Bilirubin; DBIL, Direct Bilirubin
